# Respiratory-gated (4D) contrast-enhanced FDG PET-CT for radiotherapy planning of lower oesophageal carcinoma: feasibility and impact on planning target volume

**DOI:** 10.1186/s12885-017-3659-9

**Published:** 2017-10-04

**Authors:** Andrew Scarsbrook, Gillian Ward, Patrick Murray, Rebecca Goody, Karen Marshall, Garry McDermott, Robin Prestwich, Ganesh Radhakrishna

**Affiliations:** 1 Department of Radiology, Leeds Teaching Hospitals NHS Trust, Leeds, UK; 2 Department of Nuclear Medicine, Leeds Teaching Hospitals NHS Trust, St James’s University Hospital, Level 1, Bexley Wing, Beckett Street, Leeds, LS9 7TF UK; 3 Leeds Institute of Cancer and Pathology, University of Leeds, Leeds, UK; 4 Department of Medical Physics and Engineering, Leeds Teaching Hospitals NHS Trust, Leeds, UK; 5 Department of Clinical Oncology, Leeds Teaching Hospitals NHS Trust, Leeds, UK; 6 The Christie Hospital, Wilmslow Road, Manchester, M20 4 BX UK

**Keywords:** FDG pet-Ct, Oesophageal carcinoma, Radiotherapy treatment planning, Four-dimensional CT, Target volume definition

## Abstract

**Background:**

To assess the feasibility and potential impact on target delineation of respiratory-gated (4D) contrast-enhanced ^18^Fluorine fluorodeoxyglucose (FDG) positron emission tomography - computed tomography (PET-CT), in the treatment planning position, for a prospective cohort of patients with lower third oesophageal cancer.

**Methods:**

Fifteen patients were recruited into the study. Imaging included 4D PET-CT, 3D PET-CT, endoscopic ultrasound and planning 4D CT. Target volume delineation was performed on 4D CT, 4D CT with co-registered 3D PET and 4D PET-CT. Planning target volumes (PTV) generated with 4D CT (PTV_4DCT),_ 4D CT co-registered with 3D PET-CT (PTV_3DPET4DCT)_ and 4D PET-CT (PTV_4DPETCT_) were compared with multiple positional metrics.

**Results:**

Mean PTV_4DCT_, PTV_3DPET4DCT_ and PTV_4DPETCT_ were 582.4 ± 275.1 cm^3^, 472.5 ± 193.1 cm^3^ and 480.6 ± 236.9 cm^3^ respectively (no significant difference). Median DICE similarity coefficients comparing PTV_4DCT_ with PTV_3DPET4DCT,_ PTV_4DCT_ with PTV_4DPETCT_ and PTV_3DPET4DCT_ with PTV_4DPETCT_ were 0.85 (range 0.65–0.9), 0.85 (range 0.69–0.9) and 0.88 (range 0.79–0.9) respectively. The median sensitivity index for overlap comparing PTV_4DCT_ with PTV_3DPET4DCT,_ PTV_4DCT_ with PTV_4DPETCT_ and PTV_3DPET4DCT_ with PTV_4DPETCT_ were 0.78 (range 0.65–0.9), 0.79 (range 0.65–0.9) and 0.89 (range 0.68–0.94) respectively.

**Conclusions:**

Planning 4D PET-CT is feasible with careful patient selection. PTV generated using 4D CT, 3D PET-CT and 4D PET-CT were of similar volume, however, overlap analysis demonstrated that approximately 20% of PTV_3DPETCT_ and PTV_4DPETCT_ are not included in PTV_4DCT_, leading to under-coverage of target volume and a potential geometric miss. Additionally, differences between PTV_3DPET4DCT_ and PTV_4DPETCT_ suggest a potential benefit for 4D PET-CT.

**Trial registration:**

ClinicalTrials.gov Identifier – NCT02285660 (Registered 21/10/2014).

## Background

Oesophageal cancer is the 8th commonest malignancy worldwide with approximately 456,000 cases diagnosed in 2012 [1]. Patients with locally advanced distal oesophageal cancer are increasingly treated with chemo-radiotherapy either in the neoadjuvant setting prior to definitive surgery or as stand-alone therapy [1]. Accurate target delineation, motion assessment and target localisation is a pre-requisite for high-precision radiotherapy treatment planning (RTP). Currently intravenous contrast-enhanced computed tomography (CT) in combination with endoscopic ultrasound are the standard techniques used for definition of gross tumour volume (GTV) prior to radiotherapy in oesophageal cancer [2]. A margin for microscopic extension is applied, the clinical target volume (CTV), and finally a margin for mechanical delivery uncertainties and internal organ motion relating to respiratory motion, results in a planning target volume (PTV). Contouring the GTV on conventional three-dimensional CT (3D CT) obtained during free-breathing may result in inaccurate representation of both tumour dimensions and mean tumour position relative to other organs. Four-dimensional CT (4D CT) performed with respiratory-gating, allows CT data acquired during the breathing cycle to be sub-divided into time-resolved 3D datasets (bins). The lower oesophagus moves significantly with breathing and 4D CT facilitates quantification of motion and allows patient-specific target volume delineation [3–5]. Use of 4D CT in RTP of oesophageal cancer is intended to ensure adequate coverage of the moving target volume within the radiation field and optimises normal tissue sparing compared to 3D CT [6]. 4D CT is now standard of care for RTP of lower third oesophageal cancer patients.

^18^Fluorine fluorodeoxyglucose (FDG) positron emission tomography – computed tomography (PET-CT) is firmly established in guiding optimal management of radically treatable oesophageal carcinoma [7, 8]. The role of FDG PET-CT in RTP of oesophageal carcinoma is less well developed. Preliminary studies evaluating the role of 3D PET-CT in RTP of oesophageal cancer have reported changes in target volume with potential impact on treatment planning [9–11]. Two recent studies by the same group evaluating 4D CT and 3D PET-CT in RTP of oesophageal cancer have reported that this combination of techniques impacted on target definition [12, 13]. However, motion artefacts with 3D PET-CT can reduce target contrast, overestimate lesion size and cause inaccurate assessment of standardized uptake value (SUV) [14]. Hypothetically motion management with respiratory gating to obtain a 4D PET-CT should provide additional information for RTP resulting in more consistent target definition [15]. There is a paucity of data in this clinical scenario with a single retrospective study having assessed dosimetric implications and another small prospective study evaluating the potential of 4D PET-CT for target volume delineation in oesophageal cancer, both showing promise for target volume definition [16, 17]. The impact of 4D PET-CT on PTV definition has not yet been reported in a prospective series to the best of our knowledge.

The purpose of this study was to assess the feasibility and potential impact on target delineation of contrast-enhanced 4D PET-CT acquired in the treatment planning position, for a prospective cohort of patients with lower third oesophageal cancer.

## Methods

### Study outline

This was a non-randomised prospective single centre study in patients with distal oesophageal carcinoma suitable for treatment with radiotherapy or concurrent chemo-radiotherapy (ClinicalTrials.gov identifier – NCT02285660, Registered 21/10/2014). Trial participants underwent standard-of-care imaging including 3D FDG PET-CT, endoscopic ultrasound and radiotherapy planning 4D CT. A trial-specific contrast-enhanced 4D FDG PET-CT in the treatment position with limited coverage of the lower thorax and upper abdomen was also performed. Subsequent treatment was not affected by the trial-specific PET-CT and was delivered according to institutional clinical protocols.

### Patient selection and recruitment

Inclusion criteria were as follows: Age ≥ 18 years; World Health Organization (WHO) performance status 0–2; histologically proven distal oesophageal carcinoma; clinical decision made to proceed with radiotherapy +/− concurrent chemotherapy; measurable primary tumour +/− loco-regional metastatic lymph nodes on standard-of-care imaging; able to provide fully informed written consent; able to lie flat for 1 h; not pregnant or breast feeding. Female patients of childbearing potential agreed to use effective contraception, were surgically sterile, or were post-menopausal.

Exclusion criteria included: poorly controlled diabetes; renal impairment with estimated glomerular filtration rate < 30 mL/min; allergy to iodinated contrast media.

The study was approved by the regional Research Ethics Committee (Approval Reference 11/YH/0213). All patients provided informed written consent prior to trial entry. A total of 15 patients were recruited between October 2011 and July 2014. All patients provided informed written consent prior to study entry.

### PET-CT technique

4D PET-CT scans were performed using a 64-slice GE Discovery 690 PET-CT scanner (GE Healthcare, Amersham, UK) using Real-time Position Management (RPM) respiratory-gating hardware (Varian Medical Systems Inc., Palo Alto, CA, USA), a flat couch top and laser alignment. Patients were scanned supine in the treatment position; immobilised with a wing board and both arms raised above their heads. Serum blood glucose was checked routinely and imaging was not performed in patients with a blood glucose level of >10 mmol/L. Patients fasted for at least 6 h prior to intravenous injection of 400 MBq of ^18^Fluorine-FDG. Patients were positioned on the hard-top couch with laser alignment to skin tattoos marked during standard-of-care radiotherapy planning CT 45 min’ post injection. A non-contrast CT of the treatment area (lower thorax/upper abdomen) was obtained using standard settings: 120 kV, variable mA (min 50 max 500, noise index 22.6), tube rotation time 0.4 s, pitch 1.984 with a 2.5 mm slice reconstruction. Static PET acquisition from mid thorax to upper abdomen was then performed scanning in a cranial direction with 23 slices (50% overlap) acquired over 1 min. 60-min after tracer injection a 4D respiratory-gated PET acquisition commenced scanning in a cranial direction over the same volume (20-min acquisition). Breaths per minute (BPM) were monitored during this acquisition and average breathing period in seconds (60/average BPM) was calculated. The cine acquisition parameters for 4D CT were based on average breathing period determined from the 4D PET acquisition. The 4D CT component was obtained 35 s following a bolus of 100 ml of iodinated contrast (Niopam 300, Bracco Ltd., High Wycombe, UK) injected at 2 ml/s using the following settings; 120 kV, 150 mA, tube rotation 0.4 s per rotation, pitch 1.984, 40 mm detector coverage (centred over the tumour) with a 2.5 mm helical thickness (16 images per rotation). Cine duration varied for individual patients (product of average breathing time and scanner rotation time, 0.4 s). PET images were reconstructed using a standard ordered subset expectation maximization (OSEM) algorithm with CT for attenuation correction. Both non-attenuation corrected and attenuation corrected datasets were reconstructed.

The 4D CT and 4D PET data was divided into 10 phase bins. Post-processing was used to generate an averaged 3D PET from the 4D PET scan. No co-registration was necessary as the PET and CT components were inherently registered.

### Contouring

**Fig. 1:**
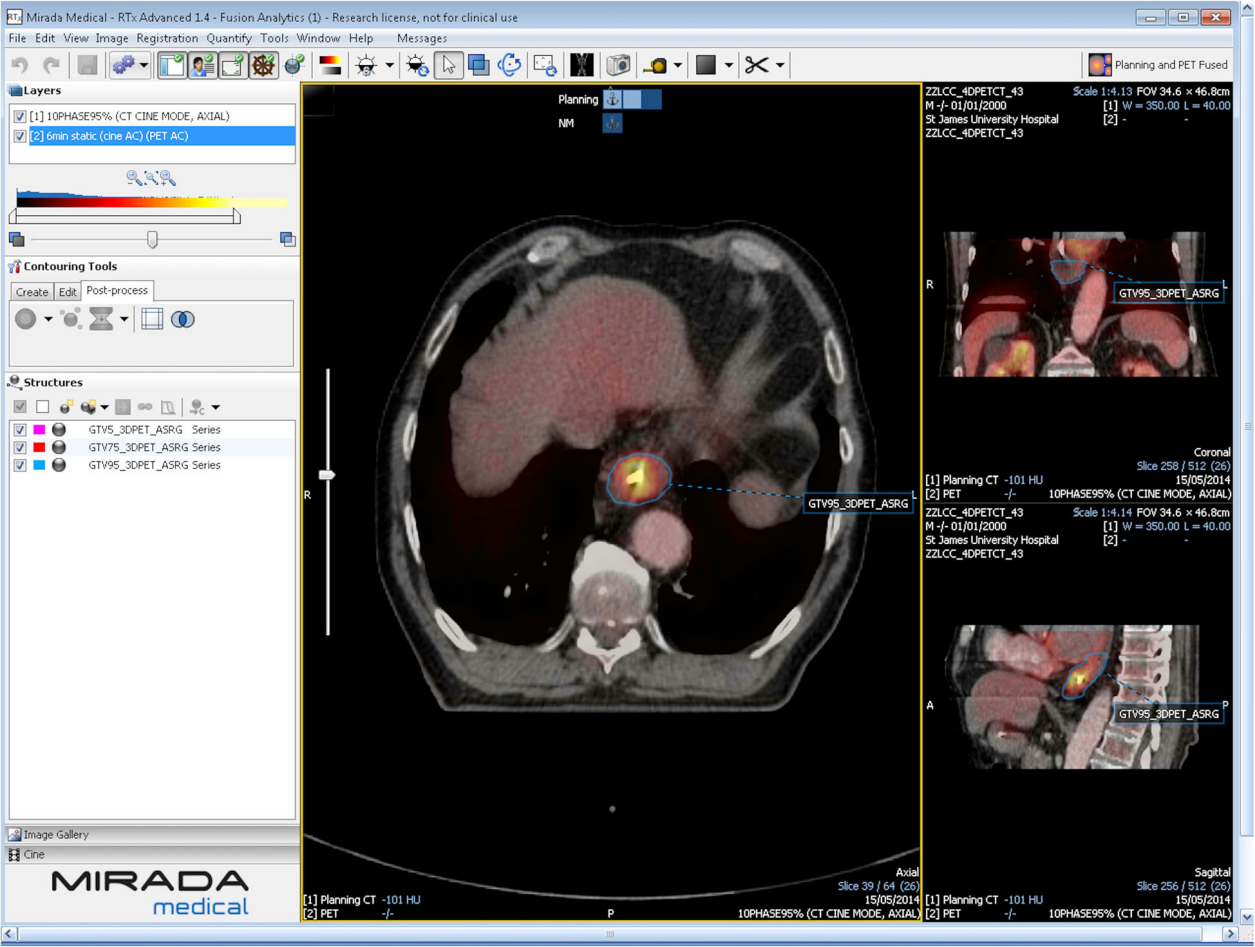
Screenshot illustrating specialised software (RTx, Mirada Medical) used to contour 4D PET and CT datasets

**Fig. 2:**
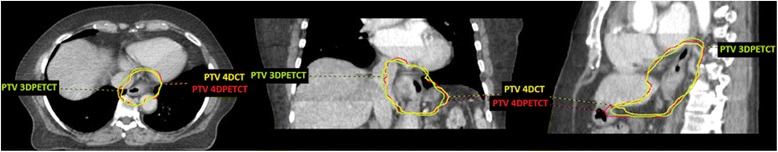
A comparison of PTV contours derived using i) 4D CT data, ii) 4D CT co-registered to 3D PET-CT and iii) 4D CT and 4D PET

Contouring was performed using specialized software (RTx, Mirada Medical, Oxford UK). PET and CT images were displayed using preset window levels and/or colour scale per a standardized institutional protocol (Fig. 1). Patients were contoured per the National Cancer Research Institute (NCRI) UK NeoSCOPE trial protocol [18]. The tumour length and outlines were derived from the diagnostic imaging which included an Endoscopic Ultrasound, contrast-enhanced CT and 3D PET-CT. The longest tumour dimension was used for outlining. The maximal length of the tumour with specific reference to an anatomical structure e.g. carina or superior aortic arch was defined on all imaging techniques. This enabled the oncologist and radiologist to identify the superior and inferior extent of the diseased oesophagus in relation to these structures; thereby allowing them to outline this segment of the entire circumference of oesophagus to be outlined. All the 4D CT and PET scans included at least one of the reference structures (e.g. superior aortic arch or carina) and therefore the target volumes were produced consistently on each of these datasets with reference to these structures [2]. Local nodal involvement was included in the target volume but more distant nodes were not. An experienced radiation oncologist contoured all target volumes with access to clinical details and standard-of-care imaging; PET-derived contours were generated by the same radiation oncologist contouring with an experienced dual-certified Nuclear Medicine Physician/Radiologist. GTVs were delineated on i) 10-phase planning 4D CT, ii) 10-phase 4D CT inherently co-registered to 3D PET-CT acquired at the same scan session and iii) 4D PET-CT. CTV was delineated and trimmed to anatomical boundaries (vertebrae, pericardium, pleura). 4D datasets from each series were used to generate an internal target volume (ITV) encompassing effects of physiological motion on the CTV. Expansion to PTV was the ITV of each series with a 5-mm margin in all directions. (Fig. 2). A minimum interval of 2 weeks was specified between delineation using each different methodology for each patient, to minimize any potential for intra-observer recall.

### Positional analysis

Five positional metrics were used to compare target volumes, calculated using ImSimQA software (v3.1.5, Oncology Systems Limited, Shrewsbury, UK): Dice index (DICE); sensitivity index (Se.Idx), inclusiveness index (Inc.Idx), centre of gravity distance (CGD) and mean distance to conformity (MDC). The Dice index produces output values ranging from 0 and 1 where 0 represents two contours with no overlap and 1 represents two contours that are perfectly overlapping [19]. The Se.Idx calculates the overlapping volume between a contour and a reference contour as a percentage of the volume of the reference volume_._ The Inc.Idx is the probability that a voxel of a contour is really a voxel of a reference contour. CGD is the distance between the geometric centres of two contours [20]. MDC is the mean of the distances between contours averaged over all positions not within the overlapping contour [20].

### Statistical analysis

Descriptive statistics (median, range) and Mann-Whitney test were used to assess for statistically significant differences between tumour volumes and lengths. Non-parametric analysis of variance (ANOVA) was used to assess the statistical significance of positional metrics between different imaging techniques (Friedman test). Stastistical analysis was performed using IBM SPSS Statistics (Version 22, IBM Corp, Amonk, NY, USA). A *p*-value <0.05 was taken as evidence of statistical significance.

## Results

### Patient characteristics

Fifteen patients were recruited to the study but only 9 underwent 4D PET-CT imaging for various reasons. Of the 6 patients who did not undergo 4D PET-CT, 3 withdrew before scanning; 2 had erratic breathing which made respiratory gating impossible and 1 patient had very low-grade tumour uptake having completed induction chemotherapy 6 weeks earlier. It was decided to discontinue data acquisition following the initial static PET for this patient. A further 2 patients who underwent 4D PET-CT had erratic breathing leading to technical difficulties with acquisition of the 4D CT component. Consequently, only 7 patients had complete datasets suitable for comparative analysis. 6 of these patients were treated with curative intent and 1 patient underwent palliative treatment. Patient characteristics are detailed in Table 1.

**Table 1:** Baseline characteristics of the 7 patients studied

Characteristic	Number
Mean age in years (range)	71 (68–74)
Histology	
*Adenocarcinoma*	6 (85.7%)
*Squamous carcinoma*	1 (14.3%)
Stage (TNM)	
T2 N0 M0	2 (28.6%)
T3 N0 M0	2 (28.6%)
T3 N1 M0	2 (28.6%)
T3 N3 M1	1 (14.3%)
Nodal disease at baseline	
Yes	3 (42.9%)
No	4 (57.1%)
Mean primary tumour SUV_max_ (Range)	12.8 (5.5–26.2)

### Volumetric comparison

Table 2 demonstrates the average delineated tumour volumes (GTV, ITV, PTV) and tumour length on the 3 different scans. PTV defined using 3D and 4D PET-CT were smaller; 3D PET-CT (PTV median 396.9, range 273.5–704.3 cm^3^): 4D PET-CT (482.1, 233.8–825.8 cm^3^) in comparison to PTV delineated on 4D CT (508.9, 267.5–907.1 cm^3^). Despite this there was no statistically significant difference between PTV volumes across the 3 methods (Mann-Whitney test). Tumour length comparison between the different modalities was also not statistically significantly different.

**Table 2:** Volumetric and length comparison of GTV, ITV and PTV structures; there were no statistically significant differences (Mann-Whitney Test) between the volumes/lengths when comparing structures from the 3D PET-CT to the 4D CT and structures from the 4D PET-CT to the 4D CT

	Volume (cm^3^)			Length (cm)		
	GTV	ITV	PTV	GTV	ITV	PTV
4D CT	108.1	271.8	528.4	5.4	10.0	10.9
3D PET-CT	82.6	239.8	472.5	6.4	10.1	11.1
*p-value*	0.89	0.95	0.95	0.52	0.8	0.7
4D PET-CT	88.3	236.0	480.6	5.1	9.6	10.6
*p-value*	0.85	0.57	0.75	0.95	0.7	0.85

*GTV* gross tumour volume, *ITV* internal target volume, *PTV* planning target volume

### Positional analysis

Median DICE similarity coefficients comparing PTV_4DCT_ with PTV_3DPET4DCT,_ PTV_4DCT_ with PTV_4DPETCT_ and PTV_3DPET4DCT_ with PTV_4DPETCT_ were 0.85 (range 0.65–0.9), 0.85 (0.69–0.9) and 0.88 (0.79–0.9) respectively. The median sensitivity index for overlap comparing PTV_3DPET4DCT_ and PTV_4DPETCT_ with PTV_4DCT_ was 0.78 (0.7–0.91) and 0.79 (0.65–0.92) respectively. The median sensitivity index for overlap comparing PTV_3DPET4DCT_ with PTV_4DPETCT_ was 0.89 (0.68–0.98). The median centre of gravity distance was 5.19 mm (1.6–27.3 mm) for the PTV_4DCT_ to PTV_4DPETCT_ structures, 3.52 mm (2.9–7.8 mm) for PTV_3DPET4DCT_ to PTV_4DPETCT_ structures and 4.97 mm (1.6–28.2 mm) for the PTV_4DCT_ to PTV_3DPET4DCT_ structures. The median mean distance to conformity was 9.15 mm (5–22.9 mm) for the PTV_3DPET4DCT_ to PTV_4DCT_ structures, 10.68 mm (5.2–22.6 mm) for the PTV_4DPETCT_ to PTV_4DCT_ and 7.41 mm (5.2–10.8 mm) for the PTV_3DPET4DCT_ to PTV_4DPETCT_.

Table 3 illustrates positional metric differences between 4D CT and 4D PET-CT PTV structures and Table 4 demonstrates the relative positional metric differences between 3D PET-CT and 4D PET-CT PTV structures. Table 5 compares the positional metrics between 4D CT and 3D PET-CT PTV structures. There was no statistically significant difference (Friedman test) between the 5 target volume positional metrics defined on each of the different CT and PET combinations (Table 6).

**Table 3:** Comparison of PTV_4DCT_ and PTV_4DPETCT_ structures

Patient	DICE	Se.Idx	Inc.Idx	CGD (mm)	MDC (mm)
1	0.9	0.91	0.89	1.73	5.23
2	0.8	0.74	0.87	9.25	12.8
3	0.89	0.85	0.94	5.19	11.45
4	0.85	0.79	0.93	1.64	10.03
5	0.69	0.65	0.74	27.29	22.56
6	0.89	0.92	0.87	2.9	5.94
7	0.82	0.77	0.88	9.33	10.68
Median	0.85	0.79	0.88	5.19	10.68

*DICE* Dice index, *Se.Idx* sensitivity index, *Inc.Idx* inclusiveness index, *CGD* centre of gravity distance, *MDC* mean distance to conformity, *SD* standard deviation

**Table 4:** Comparison of PTV_3DPET4DCT_ and PTV_4DPETCT_ structures

Patient	DICE	Se.Idx	Inc.Idx	CGD (mm)	MDC (mm)
1	0.9	0.89	0.92	2.91	5.19
2	0.9	0.92	0.88	3.25	8.35
3	0.9	0.98	0.83	6.44	6.46
4	0.88	0.83	0.93	5.49	7.41
5	0.79	0.68	0.95	3.1	7.57
6	0.82	0.94	0.73	3.52	7.4
7	0.81	0.75	0.87	7.78	10.76
Median	0.88	0.89	0.88	3.52	7.41

*DICE* Dice index, *Se.Idx* sensitivity index, *Inc.Idx* inclusiveness index, *CGD* centre of gravity distance, *MDC*mean distance to conformity, *SD* standard deviation

**Table 5:** Comparison of PTV_4DCT_ and PTV_3DPET4DCT_ structures

Patient	DICE	Se.Idx	Inc.Idx	CGD (mm)	MDC (mm)
1	0.87	0.9	0.85	4.02	5.89
2	0.77	0.7	0.86	11.23	15.44
3	0.85	0.76	0.98	4.97	10.11
4	0.9	0.88	0.92	7.03	9.15
5	0.65	0.72	0.59	28.17	22.94
6	0.85	0.78	0.94	4.19	6.8
7	0.9	0.91	0.89	1.61	5.04
Median	0.85	0.78	0.89	4.97	9.15

*DICE* Dice index, *Se.Idx* sensitivity index, *Inc.Idx* inclusiveness index, *CGD* centre of gravity distance, *MDC* mean distance to conformity, *SD* standard deviation

**Table 6:** Positional metric analysis; there were no statistically significant differences (Friedman Test) between the 5 metrics comparing PTV structures between 3D PET-CT to 4D CT, 4D PET-CT to 4D CT and 3D PET-CT to 4D PET-CT

Positional Metric	p-value
DICE	0.62
Se.Idx	0.87
Inc.Idx	0.90
CGD	0.37
MDC	0.37

*DICE* Dice index, *Se.Idx* sensitivity index, *Inc.Idx* inclusiveness index, *CGD* centre of gravity distance, *MDC* mean distance to conformity, *PTV* planning target volume

## Discussion

Respiratory motion affects imaging quality which is of direct relevance in RTP of potentially mobile tumours [21]. 4D CT guides generation of patient specific volumes accounting for physiological organ motion and is recommended in target definition protocols for lower oesophageal carcinoma [22]. Incorporating PET into the planning process has potential to improve the accuracy of target delineation but it is unclear if 4D PET-CT is superior to combining 3D PET with 4D–CT [12, 13, 15–17]. The aims of this pilot study were to assess the feasibility and impact of incorporating 4D PET-CT into the radiotherapy planning pathway of patients with lower oesophageal carcinoma.

This study has confirmed that incorporating 4D PET-CT into the RTP pathway of patients with lower oesophageal carcinoma is feasible. However, careful patient selection is important as it may not be possible in patients with an irregular breathing pattern. This prevented or significantly degraded 4D imaging in 4 patients in our study. Low-grade tumour uptake was also a limitation. The only other prospective study of 4D PET-CT in this setting reported 6/18 patients (33.3%) had imaging unsuitable for contouring for similar reasons [17]. Their study focused on optimal thresholding of PET data for GTV delineation and reported that a threshold setting of 20% standardised uptake value (SUV) or a fixed threshold of SUV 2.5 best correlated with tumour length, volume ratio and conformality index [17].

In this study 4D CT and PET data acquisitions were acquired at the same attendance with the patient in the radiotherapy treatment position with immobilization to ensure inherent co-registration. Post-processing was used to generate an averaged 3D PET from the 4D PET scan. This methodology should largely obviate any potential error introduced by registration of separately acquired CT and PET data. To the best of our knowledge, this is the first study to report positional metric comparison of tumour volumes delineated on 4D CT and 4D PET-CT in lower oesophageal carcinoma. Concordance between the contoured volumes on different studies were high with no statistical significance reached between PTV measured on each combination for the 5 positional metrics analysed, although the small sample size limits the value of statistical analysis; overlap indices were greatest between 3D PET-CT and 4D PET-CT (median DICE similarity coefficient 0.88, median sensitivity index 0.89) and slightly lower when comparing 4D CT with 3D PET-CT (median DICE similarity coefficient 0.85, median sensitivity index 0.78) and 4D CT with 4D PET-CT (median DICE similarity coefficient 0.85, median sensitivity index 0.79). Median centre of gravity distance was smallest for PTV_3DPET4DCT_ to PTV_4DPETCT_ structures (3.52 mm) and greatest for PTV_4DCT_ to PT_4DPET4DCT_ structures (5.19 mm). The median mean distance to conformity was lowest (7.41 mm) for the PTV_3DPET4DCT_ to PTV_4DPETCT_ and highest for the PTV_4DPETCT_ to PTV_4DCT_ (10.68 mm). The observed differences between PTVs are potentially clinically important; for example, the median sensitivity index of 0.79 between 4D CT and 4D PET-CT implies 21% of the PTV_4DPETCT_ is not included within the PTV_4DCT_. The use of 4D PET-CT might minimise the risk of geometric miss but this has not been confirmed and requires further evaluation.

A key unanswered question is whether 4D PET-CT provides incremental benefit compared with 4D CT and 3D PET-CT. Although positional metrics comparing PTVs generated using these methods suggested differences were small, the sensitivity index of 0.89 (implying that 11% of the PTV_4DPETCT_ was not included within PTV_3DPET4DCT_) suggests that there might be a potential additional role for the use of 4D PET-CT but the cohort is too small to draw conclusions on this. A small retrospective study of 4 patients with lower oesophageal cancer reported that target volumes delineated on inhale and exhale phase 4D PET-CT deformably co-registered with a planning CT were up to 30% smaller in 3 of 4 patients on the exhale plan with potential for dose sparing to organs at risk [16]. Conversely, a pilot study of 4D PET-CT for delineation of ITV in lung tumours reported concordance between different contoured volumes assessed using Dice coefficient and 3D PET-CT was consistently found to underestimate ITV compared to 4D PET-CT [23].

An important issue regarding use of PET in tumour volume delineation is the methodology employed to define the functional volume of interest. Several different methods have been proposed varying by tumour site and range from simplistic manual delineation to fully automated adaptive thresholding techniques [24, 25]. To date there is no clear consensus on which method is best and some nervousness about automatic delineation techniques [26]. Expert manual delineation by an experienced clinical oncologist in collaboration with a Radiologist/Nuclear Medicine Physician is established best practice [27]. In the absence of a widely accepted method in this clinical scenario, we made a pragmatic decision to manually contour PET volumes collaboratively as a Radiotherapist/Radiologist pair. Images were displayed using preset window levels and/or colour scale as per a standardized protocol which has been used in routine clinical practice for many years at our institution. Comparison of different methods for tumour volume segmentation has been previously reported in this setting albeit without pathological validation [17]. As a consequence this aspect was not a focus in this study.

PTVs delineated using 4D CT are often smaller than with 3D CT and doses to organs at risk in oesophageal cancer (lungs, liver and heart) are reduced using 4D CT which may facilitate dose escalation without significant collateral damage [28]. In this study, average tumour length was smallest on 4D PET-CT (range 5.1–10.6 cm), slightly larger (difference not statistically significant) on 4D CT (range 5.4–10.9 cm) and larger still on 3D PET-CT (range 6.4–11.1 cm). Tumour volumes were also consistently smaller on 4D PET-CT (average PTV 480.6 cm^3^) compared to 4D CT (average PTV 528.4 cm^3^), although the difference between volumes was not statistically significant. 3D CT represents a random snapshot during the breathing cycle whereas 4D CT is a selected series of snapshots at points on the breathing cycle. PET is different; 3D PET is a time-averaged image (showing motion blur covering the full breathing cycle) and 4D PET is a time-averaged image covering a phase bin where the amount of motion blur depends on degree of motion within that bin. Differences between volumes in part reflects this but also that PET and CT are demonstrating different tumour characteristics which are unlikely to inherently have the same volume but should provide complimentary information. 4D PET-CT volumes may have been smallest due to the benefit of PET and CT information but with a reduction in the motion blurring of 3D PET.

The study has limitations particularly the small cohort size and absence of histological validation of tumour volumes which is frequently the case in feasibility studies. Despite these limitations this small study has proven the feasibility of the technique and confirmed that tumour volume delineation using 4D PET-CT results in PTV differences compared with either 4D CT alone or 4D CT combined with 3D PET. Our data add to a recent literature review of the potential utility of 4D PET-CT in GTV delineation of intra-thoracic tumours which concluded that this may be the best approach currently available but warrant further investigation in future prospective studies [29]. Further work to establish if dose distribution varies for different PTVs and to evaluate outcome in a larger patient cohort with or without 4D PET-CT as part of RTP should be considered.

## Conclusions

Acquisition of a planning 4D PET-CT is feasible with careful patient selection. PTVs generated using 4D CT, 4D CT co-registered with 3D PET-CT, and 4D PET-CT were of similar volume; however, percentage overlap analysis demonstrates that approximately 20% of the PTV_3DPET4DCT_ and PTV_4DPETCT_ are not included in the PTV_4DCT_, theoretically leading to under-coverage of the target volume and a potential geometric miss. The current study is too small to draw any conclusions about clinical benefit and further investigation is warranted.

## Abbreviations

3D: Three dimensional
4D: Four dimensional (respiratory-gated)
BPM: Breaths per minute
CGD: Centre of gravity distance
CT: Computed tomography
CTV: Clinical target volume
DICE: Dice index
FDG: ^18^Fluorine-fluorodeoxyglucose
GTV: Gross tumour volume
Inc.Idx: Inclusiveness index
MDC: Mean distance to conformity
OSEM: Ordered subset expectation maximization
PET-CT: Positron emission tomography - computed tomography
PTV: Planning target volume
RPM: Real-time position management
RTP: Radiotherapy planning
Se.Idx: Sensitivity index
SUV: Standardized uptake value
WHO: World Health Organization
